# May Salivary Alpha-Amylase Level Be a Useful Tool for Assessment of the Severity of Schizophrenia and Evaluation of Therapy? A Case Report

**DOI:** 10.1155/2012/747104

**Published:** 2012-09-30

**Authors:** Masa Ieda, Tsuyoshi Miyaoka, Kiminori Kawano, Rei Wake, Takuji Inagaki, Jun Horiguchi

**Affiliations:** ^1^Department of Psychiatry, Shimane University of Medicine, 89-1 Enyacho, Izumo 6938501, Japan; ^2^Department of Psychology and Special Support Education, Shimane University of Education, 1060 Nishikawatsu, Matsue 6908504, Japan

## Abstract

*Background*. Previous studies suggested dysfunction of the autonomic nervous system (ANS) in schizophrenia patients, but the mechanism remains unclear. Recently, the measurement of salivary alpha-amylase (sAA) has been considered a useful tool for evaluating ANS, especially the sympathoadrenal medullary system. Furthermore, there was a report that patients with schizophrenia showed much higher sAA level than normal controls. *Methods*. We present the case of a 51-year-old female with catatonic schizophrenia. She needed the treatment of electroconvulsive therapy (ECT). We evaluated her sAA level and her psychiatric symptoms during the treatment. *Results*. Before ECT treatment, she showed high sAA level. Her sAA level decreased during the course of ECT, and this attenuation was accompanied by improvement of schizophrenic symptoms. *Conclusion*. We consider that measurement of the sAA level may be one of the useful biological markers for assessment of psychotic state and efficacy of treatment in patients with schizophrenia.

## 1. Introduction

 Previous studies have also shown alterations in autonomic functions using electrophysiological methods such as electrodermal measures and heart rate analysis [1–3]. These studies have demonstrated decreasing of the ANS in patients with schizophrenia.

 Recently, measurement of sAA has been thought to be a useful tool for evaluating the ANS and estimating mental stress [4–6]. An instrument to assess sAA rapidly with low invasiveness has been marketed for clinical use [7–9].

 Our colleague reported that sAA level in patients with schizophrenia was significantly higher than that in control subjects [10]. Furthermore, the correlation between sAA level and psychiatric symptoms was highly significant.

We here report the case of a female patient with catatonic schizophrenia. Before ECT treatment, she showed high sAA level. Her sAA level decreased during the course of ECT, and this attenuation was accompanied by improvement of schizophrenic symptoms. To our knowledge, this is the first published case report of longitudinal measurement of sAA levels and psychiatric symptoms in a patient with catatonic schizophrenia. 

## 2. Case Presentation

**Figure 1:**
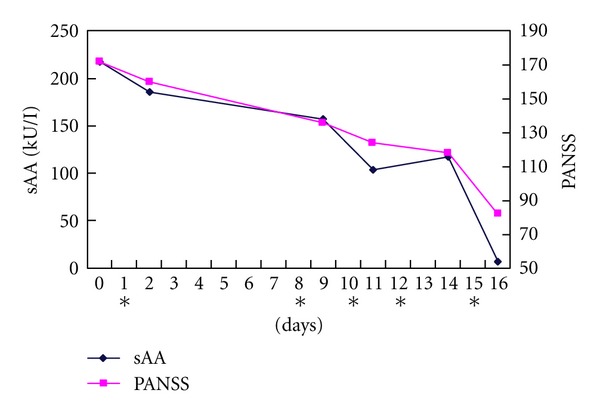
Clinical course. The day before the firstECT operation is defined as day 0. ECT operation days are denoted by an asterisk.

 The patient was a 51-year-old female. Her birth was uneventful, and she developed normally. There was no history of alcohol or drug use or epileptic seizures. Nothing was known regarding psychiatric or developmental disorders in her family. At the age of 16, she developed insomnia and auditory hallucinations and was diagnosed with schizophrenia. Since then she has had three psychiatric hospitalizations. At the age of 49, she again developed insomnia and auditory hallucinations. She intended to commit suicide because of delusions of persecution and was admitted to Shimane University Hospital. Examination of her mental state showed auditory hallucinations and delusions of persecution, catatonic stupor, autistic state, and deterioration of social functioning. Her psychiatric symptoms were evaluated by the Positive and Negative Syndrome Scale (PANSS) [11]. She suffered from lipid disorder, but other routine laboratory examinations of blood, urine, and feces were within normal limits. Electric encephalography (EEG), computed tomography (CT), and magnetic resonance imaging (MRI) of the brain showed no abnormal results. After admission, risperidone (6 to 9 mg/day) was started. At first, her psychiatric symptoms gradually decreased, and her social skills were rehabilitated. However, at the age of 51, she developed psychomotor excitement and auditory hallucinations, and finally she presented in a catatonic stupor. When she was given haloperidol (10 mg/day) and perospirone (48 mg/day) in place of the risperidone, her symptoms worsened. Therefore, modified ECT two or three times a week was started as a supplementary strategy. Around the same time, we measured her salivary amylase twice a week when there was no ECT treatment. We used a hand-held monitor (Nipro Co., Japan) to measure sAA automatically using reagent paper. Using a previously developed method [7, 8], a saliva sample was collected once in the morning (10:00–12:00 a.m.), as sAA activity shows a diurnal pattern with a steady increase in activity during the course of the day [12, 13]. Saliva was collected more than two hours after the last meal in order to eliminate the influence of food and beverage on the sAA activity [8]. She was instructed to refrain from brushing her teeth or eating for at least 60 min before the measurement and to rest in a chair or on a bed for at least 10 min [14, 15]. During the period of ECT, her prescription was not changed. After ECT was started, her symptoms gradually improved. She became almost symptom free after the seventh ECT, so the ECT was discontinued. At the same time that her symptoms improved, her sAA decreased. The clinical course is shown in Figure 1. Finally, she was transferred to another hospital for further training in social skills. The patient gave her written informed consent before the ECT treatment after the purpose and procedure had been fully explained to her.

## 3. Discussion 

 As for biomarkers, the measurement of sAA was reported to be useful for assessing mental stress [4, 5]. To date, a number of biomarkers, such as cortisol and catecholamines, have been found to reliably indicate the reactivity of physiological stress systems, for example, the hypothalamic-pituitary-adrenal (HPA) and sympathetic-adrenal-medullary (SAM) systems. In addition, previous studies that examined the response of sAA to the activity of SAM levels were correlated with increased plasma catecholamine (norepinephrine), indicating sympathetic nervous system activation [8, 12, 16]. Measurement of salivary cortisol or sAA, which can be sampled noninvasively, has been evaluated as stress biomarkers [4, 13]. 

Previous studies reported a significant increase in sAA following psychosocial stress, indicating a stress dependent activation of sAA and association of high stress levels with higher sAA levels [4, 15, 17, 18]. When sudden stressful stimuli occur, sympathetic fibers trigger the salivary gland, which secretes amylase before the gland responds to norepinephrine from the adrenal medulla; it is thought to be faster than the response to norepinephrine, and it usually occurs within minutes [19]. Cortisol is also suitable as a similar stress marker [20]. However, because the cortisol response is hormonally mediated, its reaction tends to be sluggish. sAA has the potential to become a marker of autonomic activity because salivary gland secretion is regulated by both sympathetic and parasympathetic nerves [4]. The two autonomic nerve systems work in a coordinated way to increase salivary secretion [21]. The results of previous studies of sAA reactivity to psychological stimuli have suggested it as a potential direct marker and have used it as a good measure of sympathetic-adrenal-medullary activity [4, 19, 22]. Further, a hand-held monitor was recently developed and is marketed as a noninvasive, easy-to-use, and very rapid (one minute) sAA measuring system.

 There is little evidence as to which branch (sympathetic or parasympathetic) of the autonomic nervous system is predominant in the increases in sAA during psychosocial stress. Nater et al. assessed heart rate variability (HRV) parameters during stress and reported a positive relationship between sAA and sympathetic tone [15]. Previous studies pointed out a predominant role of the sympathetic nervous system in the secretion of sAA, together with parasympathetic withdrawal under the psychosocial stress [5, 16]. Several studies of patients with schizophrenia suggested that they have markedly depressed autonomic nervous activity. Although autonomic dysregulation in patients with schizophrenia has been reported, the precise mechanisms underlying the neurobiology of the schizophrenia-related autonomic nervous system are still unclear [1, 3, 23–25]. Bär et al. showed low parasympathetic activity in patients with acute schizophrenia and suggested that schizophrenia occurred at the same time as a loss of vagal efferent activity, probably due to disturbance of the cortical-subcortical circuits modulating the autonomic nervous system [1]. Fujibayashi et al. also suggested that patients with schizophrenia possessed markedly lower autonomic nervous system activity by assessing HRV [3]. Toichi et al. revealed that the parasympathetic index was significantly decreased without significant changes in the sympathetic index and demonstrated that a psychotic state could affect the autonomic nervous system in patients with chronic schizophrenia receiving neuroleptic treatment; they also showed that a psychotic state might act as a mental stressor, suggesting a relationship between cerebral cognition and peripheral autonomic nervous function [23]. Williams et al. reported reduced activity in amygdale-medial prefrontal circuits in patients with schizophrenia [26]. This functional break of the connection between autonomic and central systems might affect brainstem neurons and the sympathoparasympathetic balance consecutively [25].

 Inagaki et al. reported that the sAA level increased significantly in the psychotic state. They suggested that greater increases in sAA might indicate more severe psychiatric symptoms and that sAA might correlate with psychiatric symptoms in patients with schizophrenia [10]. In addition, Tanaka et al. reported that there were significant differences in sAA levels between patients with major depressive disorder and healthy controls [27]. In our case, we found that the sAA level returned to normal level with amelioration of her psychiatric symptoms. We support Inagaki's report and suggest that treatment of schizophrenia improves the sympathoparasympathetic balance. We also consider that measurement of the sAA level may be one of a useful tool for assessment of the severity of schizophrenia and evaluation of therapy. Further research with additional subjects is clearly necessary because the mechanism of schizophrenia or other psychiatric disorders, especially dysfunction of the sympathoparasympathetic balance, is still poorly understood.

## References

[1] Bär KJ, Letzsch A, Jochum T, Wagner G, Greiner W, Sauer H (2005). Loss of efferent vagal activity in acute schizophrenia. *Journal of Psychiatric Research*.

[2] Schell AM, Dawson ME, Rissling A (2005). Electrodermal predictors of functional outcome and negative symptoms in schizophrenia. *Psychophysiology*.

[3] Fujibayashi M, Matsumoto T, Kishida I (2009). Autonomic nervous system activity and psychiatric severity in schizophrenia. *Psychiatry and Clinical Neurosciences*.

[4] Nater UM, Rohleder N, Gaab J (2005). Human salivary alpha-amylase reactivity in a psychosocial stress paradigm. *International Journal of Psychophysiology*.

[5] Nater UM, Rohleder N (2009). Salivary alpha-amylase as a non-invasive biomarker for the sympathetic nervous system: current state of research. *Psychoneuroendocrinology*.

[6] Inagaki T, Ieda M, Yamashia S, Tsuyoshi M, Horiguchi J (2011). Salivary alpha-amylase reactivity under psycho-physiological stress. A nonverbal communication measurement tool?. *Journal of Behavioral and Brain Science*.

[7] Yamaguchi M, Kanemori T, Kanemaru M, Takai N, Mizuno Y, Yoshida H (2004). Performance evaluation of salivary amylase activity monitor. *Biosensors and Bioelectronics*.

[8] Yamaguchi M, Deguchi M, Wakasugi J (2006). Hand-held monitor of sympathetic nervous system using salivary amylase activity and its validation by driver fatigue assessment. *Biosensors and Bioelectronics*.

[9] Uesato M, Nabeya Y, Akai T (2010). Salivary amylase activity is useful for assessing perioperative stress in response to pain in patients undergoing endoscopic submucosal dissection of gastric tumors under deep sedation. *Gastric Cancer*.

[10] Inagaki T, Miyaoka T, Okazaki S (2010). High salivary alpha-amylase levels in patients with schizophrenia: a pilot study. *Progress in Neuro-Psychopharmacology and Biological Psychiatry*.

[11] Kay SR, Fiszbein A, Opler LA (1987). The positive and negative syndrome scale (PANSS) for schizophrenia. *Schizophrenia Bulletin*.

[12] Rohleder N, Nater UM, Wolf JM, Ehlert U, Kirschbaum C (2004). Psychosocial stress-induced activation of salivary alpha-amylase: an indicator of sympathetic activity?. *Annals of the New York Academy of Sciences*.

[13] Nater UM, Rohleder N, Schlotz W, Ehlert U, Kirschbaum C (2007). Determinants of the diurnal course of salivary alpha-amylase. *Psychoneuroendocrinology*.

[14] Noto Y, Sato T, Kudo M, Kurata K, Hirota K (2005). The relationship between salivary biomarkers and state-trait anxiety inventory score under mental arithmetic stress: a pilot study. *Anesthesia & Analgesia*.

[15] Nater UM, Marca RL, Florin L (2006). Stress-induced changes in human salivary alpha-amylase activity—associations with adrenergic activity. *Psychoneuroendocrinology*.

[16] Gordis EB, Granger DA, Susman EJ, Trickett PK (2006). Asymmetry between salivary cortisol and alpha-amylase reactivity to stress: relation to aggressive behavior in adolescents. *Psychoneuroendocrinology*.

[17] Takai N, Yamaguchi M, Aragaki T, Eto K, Uchihashi K, Nishikawa Y (2004). Effect of psychological stress on the salivary cortisol and amylase levels in healthy young adults. *Archives of Oral Biology*.

[18] van Stegeren A, Rohleder N, Everaerd W, Wolf OT (2006). Salivary alpha amylase as marker for adrenergic activity during stress: effect of betablockade. *Psychoneuroendocrinology*.

[19] Skosnik PD, Chatterton RT, Swisher T, Park S (2000). Modulation of attentional inhibition by norepinephrine and cortisol after psychological stress. *International Journal of Psychophysiology*.

[20] Kirschbaum C, Hellhammer DH (1989). Salivary cortisol in psychobiological research: an overview. *Neuropsychobiology*.

[21] Proctor GB, Carpenter GH (2007). Regulation of salivary gland function by autonomic nerves. *Autonomic Neuroscience*.

[22] Shirasaki S, Fujii H, Takahashi M (2007). Correlation between salivary alpha-amylase activity and pain scale in patients with chronic pain. *Regional Anesthesia and Pain Medicine*.

[23] Toichi M, Kubota Y, Murai T (1999). The influence of psychotic states on the autonomic nervous system in schizophrenia. *International Journal of Psychophysiology*.

[24] Bär KJ, Boettger MK, Schulz S (2008). The interaction between pupil function and cardiovascular regulation in patients with acute schizophrenia. *Clinical Neurophysiology*.

[25] Bär KJ, Wernich K, Boettger S (2008). Relationship between cardiovagal modulation and psychotic state in patients with paranoid schizophrenia. *Psychiatry Research*.

[26] Williams LM, Harris AWF, Liddell BB (2004). Dysregulation of arousal and amygdala-prefontal systems in paranoid schizoprenia. *The American Journal of Psychiatry*.

[27] Tanaka Y, Ishitobi Y, Maruyama Y (2012). Salivary alpha-amylase and cortisol responsiveness following electrical stimulation stress in major depressive disorder patients. *Progress in Neuro-Psychopharmacology and Biological Psychiatry*.

